# On the Angular Distribution of the H+Li_2_ Cross Sections: a Converged Time-Independent Quantum Scattering Study

**DOI:** 10.1038/s41598-018-19233-0

**Published:** 2018-01-18

**Authors:** Henrique Vieira Rivera Vila, Luiz Antônio Ribeiro, Luiz Guilherme Machado de Macedo, Ricardo Gargano

**Affiliations:** 1 Institute of Physics, University of Brasília, P. O. Box 04455, 70.919-970 Brasília-DF, Brazil; 2 International Center for Condensed Matter Physics, University of Brasília, P. O. Box 04531, 70.919-970 Brasília, Brazil; 3 Department of Physics, Chemistry and Biology (IFM), Linköping University, SE-581 83 Linköping, Sweden; 4 Biotechnology Faculty, Institute of Biological Sciences, Federal University of Pará, 66.075-110 Belém, PA Brazil

## Abstract

A thorough time-independent quantum scattering study is performed on a benchmark potential energy surface for the H+Li_2_ reaction at the fundamental electronic state. Integral and differential cross sections are calculated along with thermal rate coefficients until convergence is reached. Our findings show that vibrational and rotational excitations of the reactant hinder reactivity, though for the latter a considerable reaction promotion was spotted as we increase the reactant rotational quantum number until the critical value of *j* = 4. Such a promotion then begins to retract, eventually becoming an actual inhibition for larger *j*. In a straightforward manner, the concept of time-independent methods implemented in this study allowed this accurate state-to-state analysis. Furthermore, a nearly isotropic behaviour of the scattering is noted to take place from the angular point of view. Remarkably, our computational protocol is ideally suited to yield converged thermal rate coefficients, revealing a non-Arrhenius pattern and showing that J-shifting approach fails to describe this particular reaction. Our results, when compared to previous and independent ones, reinforce the latest theoretical reference for future validation in the experimental field.

## Introduction

Three body reactions of the type A+BC play a central role in chemical physics as they can provide essential mechanistic informations on chemical reactions in the gas phase. Far from being trivial, they have entailed a great experimental effort since the cross molecular beam technique was first demonstrated in 1953 by Taylor and Datz^1^. The importance of studying this class of reactions can be highlighted by the 1986 Nobel Prize in Chemistry, awarded to Herschbach, Lee and Polanyi for their contributions concerning the dynamics of chemical elementary processes^2^.

Early cross beam experiments were performed for chemical reactions among hydrogen (or deuterium) atoms and (K_2_, Rb_2_ or Cs_2_) alkali homonuclear diatoms, and they all revealed considerable enthalpy variations. These observations allowed Lee, Gordon and Herschbach^3^ to infer a valuable analogy between such processes and the dynamics of the deuteron, an important projectile in nuclear physics. On the other hand, reactions involving hydrogen and lighter alkali such as lithium has become of great interest for experimentalists^4–6^, which justifies the modeling of the scattering process for the lightest alkali diatom, the Li_2_ molecule^7–9^.

In addition to hydrogen, lithium is a key element in cosmology, galatic evolution and stellar models^10^. The reaction of hydrogen and lithium yields probably the first condensed structure in the early universe^11,12^, lithium hydride, which is also of relevance for neutron shielding^13^ and hydrogen storage^14^. Moreover, the chemisorption of hydrogen on lithium clusters^11,15–17^ and the formation and depletion of LiH^18–23^ have both been extensively investigated in the theoretical field, and it is also in this context that the importance of studying the H + Li_2_ → LiH+Li reaction is inserted.

A time-independent quantum scattering study^24^ was published in 2012 at zero total angular momentum with the potential energy surface (PES) built by Maniero *et al*.^25^ for the Li_2_H ground electronic state. In this work^24^, the energetic distribution of products and the reaction probabilities for the purely vibrational/rotational excitation of the reactant molecule were investigated, as well as the thermal rate coefficients (TRC) by means of the J-shifting approach. The behaviour of the TRC at higher temperatures and the decreasing forms of the probabilistic curves appeared to agree with an expected barrierless and highly exothermic PES, which is also supported by the absence of a threshold of reactivity. This study concluded that the formation of LiH molecules which are ro-vibrationally excited by an amount of energy comparable to −Δ*H* in the particular H+Li_2_ bimolecular exchange reaction is somewhat favored, a feature observed in similar reactions involving other alkali diatoms like K_2_, Rb_2_ and Cs_2_^3^. Besides, our research group also investigated the isotopic effects for the H+Li_2_ reaction when hydrogen is substituted by muonium, deuterium or tritium, and we found that both quasi-classical^26^ and quantum^27^ results came to terms with the fact that the higher the isotope mass, the greater the cross section.

In 2014, Song *et al*.^28^ published a PES with 3726 points calculated using the multireference configuration interaction (MRCI) method, and they subsequently investigated the integral cross sections (ICS)^29^ and also the integral Coriolis coupling cross sections^30^. Gao *et al*. found in these studies that the Li_2_ rotation hinders the reaction^29^ and that the Coriolis coupling effect plays an important role in the H+Li_2_ $({\rm{X}}{{}^{1}{\rm{\Sigma }}}_{g}^{+})$ process^30^. Recently, Yuan *et al*.^31^ constructed a PES with about 30000 points employing a MRCI-F12 method and they observed deviations for the low collision energy range when compared to results from Vila *et al*.^24^, probably on account of the J-shifting approach and of the different PES adopted by the latter. Still in the time-dependent formalism and on the same PES proposed in ref.^28^, Zhu, Dong and Li^32^ employed the wave packet method with a second order split operator in order to obtain state-to-state resolved quantities, having observed that forward and backward scattering signals peaked at the two extreme angles.

The purpose of this work is to continue the H+Li_2_ time-independent quantum scattering investigations, now considering as many *J* > 0 as necessary to reach sufficient convergence on the cross sections. The reaction TRC are also calculated, and our results are qualitative and quantitatively compared to previous ones. To this end, this paper is organized as follows: while Sec. II briefly exposes the methodology employed, Sec. III brings some comments on the convergence criteria adopted in our calculations. Results are given in Sec. IV, followed by final remarks in Sec. V.

## Methodology

The ABC program^33^ solves the time-independent nuclear Schrödinger equation for an atom-diatom reaction employing the coupled channel (CC) method^34^, facing up the coordinate problem by simultaneously expanding the wave function in the Delves hyperspherical coordinates of the different arrangement channels *τ* = 1, 2, 3. Written in these coordinates, the nuclear hamiltonian assumes the form:

1 $$H=\,-\,\frac{{\hslash }^{2}}{2\mu {\rho }^{5}}\frac{\partial }{\partial \rho }{\rho }^{5}\frac{\partial }{\partial \rho }+{H}_{ad},$$

where *μ* is the three-body reduced mass, *ρ* is the hyperradius, and *H*_*ad*_ is the adiabatic term^35^. Therefore, if we denote by *η* and *γ* the two Delves angles, *ϕ*, *θ* and *ψ* the three Euler angles, *J* the total angular momentum, *M* and *K* its projections in the Space Frame and Body Frame coordinate systems, *v* and *j* the asymptotic vibrational and rotational quantum numbers, a convenient way to span the nuclear wave funcion ***χ*** comes in terms of the eigenfunctions of *H*_*ad*_ with coefficients *g* to be determined:

2 $${\chi }^{JM}=\sum _{\tau {v}_{\tau }{j}_{\tau }{K}_{\tau }}{g}_{\tau {v}_{\tau }{j}_{\tau }{K}_{\tau }}(\rho \mathrm{)\ }{B}_{\tau {v}_{\tau }{j}_{\tau }{K}_{\tau }}^{JM}(\rho ,{\varphi }_{\tau },{\theta }_{\tau },{\psi }_{\tau },{\gamma }_{\tau },{\eta }_{\tau }\mathrm{).}$$

The basis functions *B* are conceived to obey a relation written in terms of the matrix elements of the Wigner rotation operator *D*, the spherical harmonics *Y* and the “vibrational” functions *φ*:

3 $${B}_{\tau {v}_{\tau }{j}_{\tau }{K}_{\tau }}^{JM}=\frac{{D}_{M{K}_{\tau }}^{J}({\varphi }_{\tau },{\theta }_{\tau },0){Y}_{{j}_{\tau }{K}_{\tau }}({\gamma }_{\tau },{\psi }_{\tau }){\phi }_{{v}_{\tau }}({\eta }_{\tau })}{{\rho }^{5/2}\,\sin \,{\eta }_{\tau }\,\cos \,{\eta }_{\tau }}.$$

As far as basis set convergence is concerned, ABC requires that we feed it with suitable input parameters (henceforth denoted like this), and the ones that mainly account for different *B* functions in Eq. (2) are emax, jmax and kmax, which respectively label the internal energy below which all open channels are considered, the maximum rotational and the maximum helicity quantum numbers.

The first major task performed by the program is then the basis set determination. In this step, ABC segments the hyperradius interval in mtr equally spaced grids until *ρ* = rmax. The *η*-dependent part of the hamiltonian is solved within each segment yielding the *φ* functions by means of a finite difference method, using as reference potentials the diatomic ones of each arrangement on the surface of the hypersphere.

Secondly, ABC proceeds to the calculation of the overlap and (potential/kinetical) coupling matrix elements:

4 $${O}_{\tau ^{\prime} {v^{\prime} }_{\tau }{j^{\prime} }_{\tau }{K^{\prime} }_{\tau }}^{\tau {v}_{\tau }{j}_{\tau }{K}_{\tau }}=\langle {B}_{\tau ^{\prime} {v^{\prime} }_{\tau }{j^{\prime} }_{\tau }{K^{\prime} }_{\tau }}^{JM}|{B}_{\tau {v}_{\tau }{j}_{\tau }{K}_{\tau }}^{JM}\rangle $$

and

5 $${U}_{\tau ^{\prime} {v^{\prime} }_{\tau }{j^{\prime} }_{\tau }{K^{\prime} }_{\tau }}^{\tau {v}_{\tau }{j}_{\tau }{K}_{\tau }}=\langle {B}_{\tau ^{\prime} {v^{\prime} }_{\tau }{j^{\prime} }_{\tau }{K^{\prime} }_{\tau }}^{JM}|\frac{2\mu }{{\hslash }^{2}}({H}_{ad}-E)-\frac{1}{4{\rho }^{2}}|{B}_{\tau {v}_{\tau }{j}_{\tau }{K}_{\tau }}^{JM}\rangle ,$$

which appear in the CC equations:

6 $$\frac{{d}^{2}{\boldsymbol{g}}}{d{\rho }^{2}}={{\boldsymbol{O}}}^{-1}{\boldsymbol{Ug}},$$

solved in the program by a constant reference potential log derivative algorithm^36^ so that the coefficient matrix ***g*** becomes determined.

This means that after integrations are performed in each sector for the evaluation of the ***O*** and ***U*** matrices by using Gauss-Legendre quadratures for *γ*, trapezoidal rules for *η* and analytical integrations for the Euler angles, the nuclear wave function is then matched between neighbour sectors, being propagated until the asymptotic value of the hyperradius is reached. It must be stressed that the coordinate system is switched from hyperspherical to Jacobi’s as the propagation moves away from the strong interaction domain in order to save computing time.

Finally, scattering boundary conditions are applied by the program and the parity-adapted S-matrix elements are then given in the output files for a previously specified (*J*, *P*, *p*) triple, where *P* and *p* label the triatomic and the diatomic parity eigenvalues. As parity is preserved both in the interchannel matrix elements and in the asymptotic solutions, the calculations are straightforwardly decoupled so that each different triple requires an independent program run.

Gathering sufficiently many outputs to ensure *J*-convergence, these matrix elements can be used to yield any observable property of the reaction. Nevertheless, prior to obtaining that, we need to convert them into helicity-representation S-matrix elements by means of the following equations^33^:

7 $${S}_{n^{\prime} k^{\prime} ,nk}^{J}={S}_{n^{\prime} -k^{\prime} ,n-k}^{J}=\sqrt{\frac{(1+{\delta }_{k^{\prime} 0})(1+{\delta }_{k0})}{2}}[{S}_{n^{\prime} k^{\prime} ,nk}^{J,P=+1}+{S}_{n^{\prime} k^{\prime} ,nk}^{J,P=-1}]$$

and

8 $${S}_{n^{\prime} -k^{\prime} ,nk}^{J}={S}_{n^{\prime} k^{\prime} ,n-k}^{J}={(-1)}^{J}\sqrt{\frac{(1+{\delta }_{k^{\prime} 0})(1+{\delta }_{k0})}{2}}[{S}_{n^{\prime} k^{\prime} ,nk}^{J,P=+1}-{S}_{n^{\prime} k^{\prime} ,nk}^{J,P=-1}],$$

where *n* and *n*′ are composite indices for initial *τ v j* and final *τ*′ *v*′ *j*′ states. Restrictions are made so that 0 ≤ *k* ≤ *min*(*J*, *j*, kmax) and 0 ≤ *k*′ ≤ *min*(*J*, *j*′, kmax).

This way, we are able to compute the differential cross sections^37^:

9 $$\begin{array}{rcl}\frac{d{{\rm{\sigma }}}_{n}}{d{\rm{\Omega }}}(\theta ,E) & = & \frac{{\hslash }^{2}}{8{\mu }_{H+L{i}_{2}}{E}_{col}\mathrm{(2}j+\mathrm{1)}}\sum _{v^{\prime} }\sum _{j^{\prime} }\sum _{k^{\prime} =-min(j^{\prime} ,\mathrm{kmax})}^{min(j^{\prime} ,\mathrm{kmax})}\sum _{k=\,-min(j,\mathrm{kmax})}^{min(j,\mathrm{kmax})}{|{f}_{nk,n^{\prime} k^{\prime} }(\theta ,E)|}^{2}\\ {f}_{nk,n^{\prime} k^{\prime} }(\theta \mathrm{,\ }E) & = & \sum _{J=max(|k|,|k^{\prime} |)}^{{J}_{max}}\mathrm{(2}J+\mathrm{1)}{d}_{k^{\prime} k}^{J}(\theta ){S}_{n^{\prime} k^{\prime} ,nk}^{J}(E)\end{array}$$

and the integral cross sections^38^:

10 $${\sigma }_{n}(E)=\frac{\pi {\hslash }^{{2}}}{2{\mu }_{H+L{i}_{2}}{E}_{col}(2j+1)}\sum _{v^{\prime} }\sum _{j^{\prime} }\sum _{k^{\prime} =-{\min }(j^{\prime} ,\mathrm{kmax})}^{{\min }(j^{\prime} ,\mathrm{kmax})}\sum _{k=-{\min }(j,\mathrm{kmax})}^{{\min }(j,\mathrm{kmax})}\sum _{J=0}^{{J}_{{\max }}}{|{S}_{n^{\prime} k^{\prime} ,nk}^{J}(E)|}^{2},$$

where *E*_*col*_ stands for the collision energy, ${\mu }_{H+L{i}_{2}}$ for the reactant reduced mass and ${d}_{k^{\prime} k}^{J}(\theta )$ for the Wigner small d-matrix elements.

The computation of the thermal rate coefficients also becomes possible^39^ once we are able to truncate the following series for the cumulative reaction probabilities:

11 $$N(E)=\sum _{J=0}^{{J}_{{\max }}}(2J+1){N}^{J}(E),$$

which can be expressed in terms of the different (*J*, *P*, *p*)-output and of the H+Li_2_ nuclear-spin weights (*w*_*p*=+1_ = 6 for even and *w*_*p*=−1_ = 10 for odd diatomic parities^40^) if we take into account that:

12 $$\begin{array}{rcl}{N}^{J}(E) & = & {w}_{p=+1}[{N}^{J,P=+1,p=+1}(E)+{N}^{J,P=-1,p=+1}(E)]\\  &  & +{w}_{p=-1}[{N}^{J,P=+1,p=-1}(E)+{N}^{J,P=-1,p=-1}(E)]\\ {N}^{J,P,p}(E) & = & \sum _{nk}\sum _{n^{\prime} k^{\prime} }|{S}_{n^{\prime} k^{\prime} ,nk}^{J,P,p}(E{)|}^{2}.\end{array}$$

Then, writing *Q*_*R*_ (translational and ro-vibrational reactant partition function) as:

13 $$\begin{array}{rcl}{Q}_{R} & = & {Q}_{trans}\cdot {Q}_{rovib},\\ {Q}_{trans} & = & {[\frac{2\pi {\mu }_{H,L{i}_{2}}{k}_{B}T}{{h}^{2}}]}^{3/2}\end{array}$$

and

$${Q}_{rovib}=\sum _{vj}{w}_{p}(2j+1){e}^{-E(v,j)/{k}_{B}T},$$

the TRC can be calculated by means of the following expression:

14 $$k(T)=\frac{1}{h{Q}_{R}}{\int }_{0}^{\infty }{e}^{-E/{k}_{B}T}N(E)dE\mathrm{\ .}$$

## Input and Convergence

### Potential Energy Surface

The reliability of the results yielded by the study performed here is strongly connected to the quality of the input employed. By this we mean that no choice on any parameter used should be seen as pure randomness or mere convenience.

That said, we begin this section by emphasizing that the PES, which enters the problem as a part of the adiabatic term of Eq. (1), was chosen as the one published in ref.^25^, where 394 non-equivalent electronic energies for the Li_2_H system in the fundamental state were computed using a norm-conserving pseudo-potential to represent the lithium core and a 6–311 G (2df, 2pd) basis set to perform a full configuration interaction (CI) calculation. These specific 394 ab-initio points were taken in the most important parts of the interaction, in order to describe the electronic part of the H+Li_2_ reaction avoiding under or overcompleteness that would compromise the expected behaviour elsewhere.

Bond-Order (BO) polynomials of degree 8 for two- and three-body terms were then used to yield the analytical representation, which resulted in a root mean square deviation *δ* of about 1 kcal/mol. It is worth stressing that only relevant ab-initio energies were taken into account, as the authors did not resort to placing numerous points (in the asymptotic regions, for instance) just to grandstand about the low *δ*-value of the surface fitting without a relevant (and corresponding) gain in quality. This statement can be underpined by the satisfactorily good comparison among properties extracted from the PES and their equivalent in the literature: geometries, energies, ro-vibrational frequencies, enthalpy variation and other characteristics were well reproduced by this PES, being supported by independent theoretical and experimental data available in the literature^41–47^.

Table 1 exemplifies what we mean, regardless of minor divergences that arise on account of the different methodologies involved. For instance, Song and collaborators^28^ have calculated 3726 ab initio energies at the MRCI level using the full valence complete active space (FVCAS) reference function and the Dunning’s V5Z basis set. A correction was then implemented by the double many-body expansion-scaled external correlation (DMBE-SEC) method. Conversely, Yuan *et al*.^31^ employed the MRCI-F12 method with the aug-cc-pVTZ basis set to calculate 30000 electronic energies, performing next a surface fitting based on neural networks.

**Table 1:** Comparison among data extracted from different PES in the literature and experimental results: equilibrium distances, dissociation energies, spectroscopic constants, angles and other properties obtained for the Li_2_/LiH diatoms and for the three-body interaction region in the global minimum configuration.

	Ref.^25^	Ref.^28^	Ref.^31^	Experimental
number of ab-initio points root	394	3726	30000	
mean square deviation (kcal/mol)	1	0.636	0.299	
		Li_2_ (X ^1^Σ*g*)		
*R*_*e*_ (bohr)	5.0512	5.0877	5.0531	5.0512^41^
*D*_*e*_ (kcal/mol)	24.438	24.445	24.398	24.444^44^
*ω*_*e*_ (cm^−1^)	351.48	353.528	351.49	351.4^41^
*ω*_*e*_*x*_*e*_ (cm^−1^)	2.652	2.655		2.595^41^
		LiH (X ^1^Σ)		
*R*_*e*_ (bohr)	3.151	3.0336	3.0160	3.0160^41^
*D*_*e*_ (kcal/mol)	58.099	58.113	58.0203	58.112^43^
*ω*_*e*_ (cm^−1^)	1405.7	1387.886	1410.82	1405.6^41^
*ω*_*e*_*x*_*e*_ (cm^−1^)	21.2	23.693		23.2^41^
		Li_2_H (X^2^A’)		
*R*_*e*_ (Li-Li) (bohr)	4.7366	4.7621	4.7205	4.7621^47^
*R*_*e*_ (Li-H) (bohr)	3.2212	3.2474	3.2201	3.2409^47^
$\widehat{HLiLi}$ (°)	42.675	42.843	42.813	42.708^47^
Depth of the potential minimum relatively to the H + Li + Li asymptote (kcal/mol)	86.9	87.91	85.16	87.9 ± 3^46^

Even though the authors from ref.^25^ make use of considerably fewer ab-initio points than the other referred researchers, it cannot be inferred that the PES we employed here is inferior in any sense: when compared to the PES from ref.^28^, the former shows slightly better diatomic results with respect to the experimental data available, and a little less refined agreement as far as the triatomic minimum configuration is concerned; as for the PES from ref.^31^, on the other hand, both diatomic and triatomic comparisons favor ref.^25^. It should be emphasized, however, that all three PES accurately represent the title reaction, and results derived from them should be compared whenever possible. Two of the most remarkable features of the scattering process, independently obtained by all three studies, are its essentially barrierless behaviour and its high exothermicity (around 34 kcal/mol).

Be that as it may, when it comes to the practical situation in which the Li_2_H PES must be embedded as a subroutine in the ABC time-independent calculations, two aspects must be highlighted. First, the use of more elaborate analytical forms like the ones comprised in refs^28,31^ might have led to a mounting complexity at the expense of computational resources, which have already been exhaustively explored with the use of the BO polynomials of ref.^25^. Second, the fitted expansion parameters of the^28,31^ potential energy surfaces are not available from the literature the way^25^ is, making them impossible to be employed unless the interested reader is granted access to the data upon request.

### ABC Input Parameters

Moving on with the discussion to the ABC input described in ref.^33^, we provided the program with the parameters shown in Table 2. For different choices on the (*J*, *P*, *p*) triple, we had to make sure that the designated values for rmax and mtr were such that: a) the scattering would be studied until the asymptotic behaviour manifests itself; and b) the size of the grids would allow us to treat the sectors with an adiabatic approach. As a matter of fact, attention has been paid to the adiabatic curves in the manner described by ref.^24^ for different program executions, and both conclusions could be drawn because these curves: a) stabilize before the maximum hyperradius considered is reached; and b) they experience sufficiently smooth variations as we move from one sector to another. Also, fixing rmax and mtr at 25 a_0_ and 300 proved to be convenient for us since we carefully inspected the impact to the final convergence of the cross sections of further varying these parameters, especially in the lowest collision energy studied (0.21 meV for the Li_2_(*v* = 0, *j* = 0) state), conditions under which changing rmax and mtr simultaneously to 30 a_0_ and 400 represented only a decrease of less than 1.9 % in the ICS.

**Table 2:** ABC input parameters used for the H + Li _2_ reaction.

Parameter	Meaning
mass = 1, 7, 7	Masses of the atoms in atomic mass units.
jtot = 0, 1, 2, ..., 80	Total angular momentum *J*.
ipar = ±1	Triatomic parity eigenvalue *P*.
jpar = ±1	Diatomic parity eigenvalue *p* = (−1)^*j*^.
emax = 0.68	Maximum internal energy in any channel (in eV).
jmax = 35	Maximum rotational quantum number of any channel (*j* or *j*′).
kmax = 4	Helicity truncation parameter.
rmax = 25.0	Maximum hyperradius *ρ* (in a 0).
mtr = 300	Number of log derivative sectors.
enrg = 0.022	Initial total energy (in eV).
dnrg = 0.02	Total energy increment (in eV).
nnrg = 20	Number of different total energies.
nout = 3	Maximum value of *v* for the output.
jout = 15	Maximum value of *j* for the output.

Next, we had to limit the emax, jmax and kmax parameters as they dictate how robust our basis set should be, keeping in mind that the bigger they are, the longer calculations will take and the more they will cost in computational terms. Considering that we are interested in the low collision energy range (up until 0.4 eV), that for early calculations with *J* = 0 the choice on the first two parameters proved to be well suited for the problem, and that for linear reactions kmax does not need to be way greater than zero, we investigated the effects of modifying each and every single one of these quantities independently.

**Figure 1:**
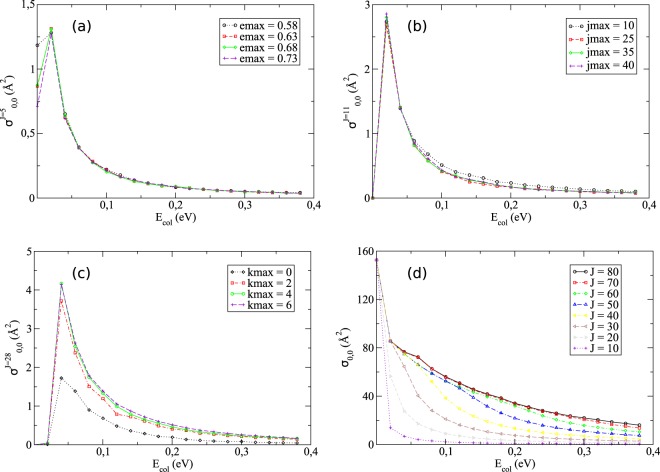
Convergence tests performed on the H+Li_2_(*v* = 0, *j* = 0) → LiH + Li integral cross sections for: (**a**) the partial contribution of *J* = 5 fixing jmax = 35 and kmax = 4; (**b**) the partial contribution of *J* = 11 fixing emax = 0.68 and kmax = 4; (**c**) the partial contribution of *J* = 28 fixing emax = 0.68 and jmax = 35; and (**d**) the sum over all total angular momentum contributions from 0 to *J* in each curve (emax = 0.68, jmax = 35 and kmax = 4).

To this end, we first varied the emax parameter keeping the other two constant so to visualize the consequence on the partial contributions of the integral cross sections. Figure 1(a) displays one particular case for a 0.05 eV increment on the maximum internal energy ranging from 0.58 to 0.73 eV. Out of curiosity, the number of channels considered for the (*J* = 5, *P* =−1, *p* = +1) calculation jumps from 3286 to 3853 in these limits, being equal to 3664 for emax = 0.68. Performed on an IBM P750 machine using Power7 processors with 3.55 GHz of frequency (containing cores of 128 GB RAM memory and 908.8 GFlops of theoretical performance), the calculations for emax = 0.68 took almost 12 days.

From the analysis of Fig. 1(a), we see that once again emax = 0.68 is a good choice not only for *J* = 0, as little variation is identified among the different curves for the particular case in which *J* = 5 (a feature also remarked for other non-trivial *J*-values).

Verifying now the outcome of the variation on jmax once emax and kmax are fixed, we end up with satisfactory convergence for jmax = 35, as Fig. 1(b) exemplifies for a specific situation (*J* = 11). It is of utter importance to stress that other partial contributions on the integral cross sections were also duly studied, although only one of them is represented here.

Passing to the same analysis for kmax, we observe that very little is changed when we increase this parameter from 2 to 4, and even less when we take into account kmax = 6, so it would be pointless to go way beyond that limit. Figure 1(c) depicts one such example for a given *J*-value (*J* = 28). For our purposes, thus, considering the helicity truncation parameter kmax = 4 suffices to yield the desired results.

The last parameter that remains to be commented on has to do with how far we must go with the *J*-values until some sort of criterion is met. On that subject, we verified that working with *J*_*max*_ = 80, as suggested by ref.^29^, leads to reasonable convergence, for (*σ*^*J*=80^ − *σ*^*J*=79^)/(*σ*^*J*=80^) <0.32%. This is a conclusion that is brought graphically by Fig. 1(d): it was already expected that a huge amount of *J*-values would be required for the title reaction, owing to its own peculiarities, such as high exothermicity directly related to a large number of basis functions needed to span the nuclear wave function. This almost unfeasible approach helps to explain why it took so long for a full time-independent quantum scattering approach to happen.

A particular topic that deserves our attention, however, has to do with the reliability of ABC in the case of indirect reactions such as the title one, in which a deep potential well involving a long-lived intermediate complex separates reactants and products. In other words, questionings may arise on the suitability of the diatomic vibrational functions of the three arrangements to expand the surface functions, what can be understood as the use of a constant reference potential instead of the true triatomic potential for a given value of the hyperradius. In this concern, while this doubtfulness may seem valid, it must be stressed that extra care was paid so to consider a sufficiently big amount of eigenstates to span the nuclear wave function in order to surmount this apparent shortcoming. This way, the present application of the methodology evidences good results when compared to different studies, as will be shown in the next section. Other successful ABC calculations for scatterings proceeding over deep potential wells were already reported in the literature and can be found in refs^48–52^.

## Results and Discussions

### Integral Cross Sections

**Figure 2:**
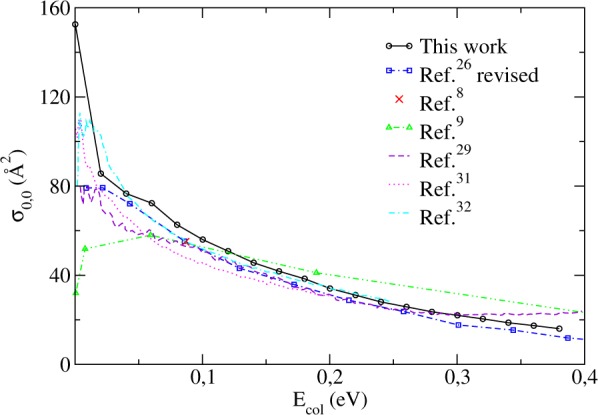
Integral cross sections as a function of the collision energy for the H+Li_2_(*v* = 0, *j* = 0) → LiH + Li reaction.

Having run the ABC program as indicated in the previous section, we came up with numerous blocks of state-resolved S-matrix elements in the parity-adapted representation ${S}_{n^{\prime} k^{\prime} ,nk}^{J,P,p}(E)$. Then, aiming to obtain the integral cross sections, we had to externally develop a FORTRAN code that would basically perform the transformations of Eqs (7) and (8) prior to proceeding to the summation described by Eq. (10). For further details on this subject, please refer to the Supplemental Material. Selecting first the initial state (*v* = 0, *j* = 0) of the reactant for the sake of simplicity and in order to compare our results with others available in the literature (yielded by the application of different methods), we ended up with the black circles plotted in Fig. 2.

On this subject, it can be stated that sufficiently close theoretical agreement has been reached with the minor exception of the original data published by ref.^26^, which brought results laying way below the average, although the use of quasi-classical trajectories (QCT) might have seemed advisable given that the characteristics of the Li_2_ H PES did not suggest a considerable tunneling contribution. Despite the endorsement by preliminary QCT calculations performed in ref.^25^, the overall discrepancy of about 10 times less the order of magnitude of all other available results urged us to recheck it for possible errors. After a thorough debugging of the original code gently provided to us by the authors, an improper reading procedure at the level of the interpretation of the PES subroutine (causing a swapped identification of the BO coefficients) has been spotted and corrected. The Revised QCT curve now agrees with the others, as it should.

Furthermore, for those works relying on distinct PES, we sort of expected in advance that the slightest difference regarding mainly the three-body high interaction region – such as the presence or not of a barrier, the precise depth of the potential well, or even the geometries and frequencies in the minimum energy path, for example – or the nature of the theoretical scattering formalism involved would entail very unrelated results, leading perhaps to important numerical discrepancies, which fortunately was not the case. As far as quantum scattering methods are concerned, however, it can be claimed that while time-dependent calculations (refs^29,31,32^.) offer an easier interpretation to the microscopic mechanisms of the reaction when compared to the time-independent studies, some problems may arise in the former when propagating initial wave packets with low collision energies, thus requiring extra care^53,54^. The combined analyses of these independent studies will serve as future reference for comparison, ultimately being subject to experimental validation or confrontation.

**Figure 3:**
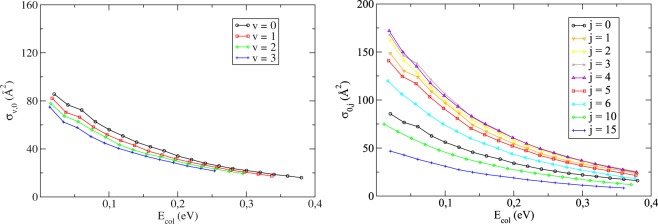
Integral cross sections (summed over all product states) as a function of the collision energy for the purely vibrational (left) or rotational (right) excitation of the H+Li_2_(*v*, *j*) → LiH + Li reaction.

Examining now the effects due to the purely vibrational/rotational excitation in Fig. 3, we see that for both cases the H+Li_2_ reaction is more and more inhibited as the *v* or *j*-values are increased. For the rotational case, however, we identified a considerable reaction promotion that augments until *j* = 4 is reached, and then begins to retract, eventually becoming an actual inhibition for larger *j*. Additionally, all curves reveal a monotonically decreasing behaviour, a fact that is intimately related (as it should be) to the highly exothermic and barrierless nature of the scattering. The overall conclusion that the reactivity is hindered for both types of excitation and the general trend of the integral cross sections for different initial states is consistent with the latest publications in the literature^24,29^.

**Figure 4:**
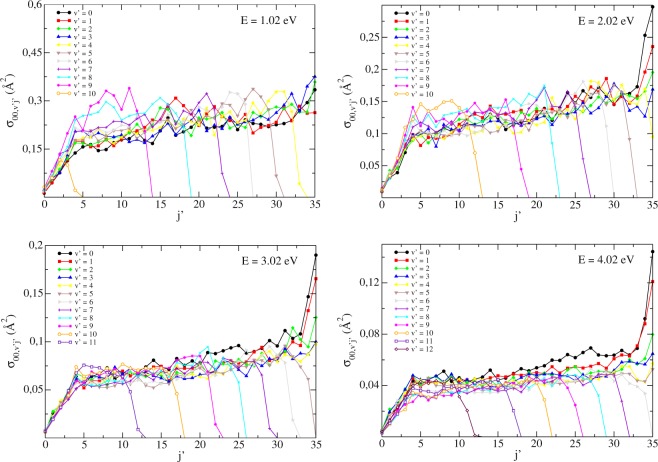
State-to-state ICS at different total energies *E* for the (*v* = 0, *j* = 0) initial state.

In order to better elucidate the dynamics involved, we also investigated the state-to-state cross sections aiming to account for the product distribution with respect to the *v*′ and *j*′ quantum numbers, as shown in Fig. 4 for the (*v* = 0, *j* = 0) initial state. From this analysis, basically two patterns also identified in ref.^32^ emerge. First, for a given vibrational (rotational) quantum number, we see that the reaction cross sections tend to grow until the *j*′-values (*v*′-values) begin to relate to prohibitive ro-vibrational energies, thus experiencing a sudden drop from that point on and revealing that the product states most likely to be formed are those which incorporate the majority of the total energy in the form of diatomic excitation, reinforcing the preliminary conclusions drawn by ref.^24^ on that matter. Second, the globally dense rotational distribution seen in Fig. 4 strongly suggests that the reaction mechanism is substantially statistical.

### Differential Cross Sections

As far as the H+Li_2_ differential cross sections are concerned, they can promptly be evaluated once we proceed analogously as before, where instead of Eq. (10) for the integral cross sections, we now take into account Eq. (9).

This calculation, of great value to experimentalists as they can easily detect the angular dependence of the scattering cross sections, yields directly from the time-independent formalism employed by ABC, provided that we use the most suitable expressions for the Wigner small d-matrix elements ${d}_{k^{\prime} k}^{J}(\theta )$ as the *J* values are increased.

This unusual remark is based on recent investigations on the referred theme, according to which incorrect divergent behaviour begins to be evidenced when *J* becomes ≫1 if we use the most commonly known expressions for ${d}_{k^{\prime} k}^{J}(\theta )$. In our case, for instance, a sudden inconsistency near *θ* = *π*/2 manifested itself in the form of a large peak for *J* > 56 with the application of the following equation^55^:

15 $$\begin{array}{rcl}{d}_{k^{\prime} k}^{J}(\beta ) & = & \sqrt{(J+k)!(J-k)!(J+k^{\prime} )!(J-k^{\prime} )!}\\  &  & \sum _{t}\frac{{(-1)}^{t}{[\cos (\beta /2)]}^{2J+k-k^{\prime} -2t}{[\sin (\beta /2)]}^{2t+k^{\prime} -k}}{(J+k-t)!(J-k^{\prime} -t)!t!(t+k^{\prime} -k)!},\end{array}$$

where the sum takes place for all values of *t* that do not lead to negative factorials.

It is argumented that a serious loss of precision happens because of the inclusion of large numbers that exceed the floating-point precision for Wigner’s original formula (Eq. 15) or because of severe numerical instability in the case of high spin for recurrence relations. An example of a very straightforward scheme based upon recurrence uses 3 such relations in order to determine ${d}_{k^{\prime} k}^{J}(\theta )$ provided that lower order terms have already been calculated:

16 $$\begin{array}{rcl}{d}_{k^{\prime} ,k}^{J}(\beta ) & = & {\cos }^{2}(\beta /2)\sqrt{\frac{(J+k)(J+k-1)}{(J+k^{\prime} )(J+k^{\prime} -1)}}{d}_{k^{\prime} -1,k-1}^{J-1}(\beta )\\  &  & -2\,\sin (\beta /2)\cos (\beta /2)\sqrt{\frac{(J+k)(J-k)}{(J+k^{\prime} )(J+k^{\prime} -1)}}{d}_{k^{\prime} -1,k}^{J-1}(\beta )\\  &  & +{\sin }^{2}(\beta /2)\sqrt{\frac{(J-k)(J-k-1)}{(J+k^{\prime} )(J+k^{\prime} -1)}}{d}_{k^{\prime} -1,k+1}^{J-1}(\beta )\end{array}$$

17 $$\begin{array}{rcl}{d}_{k^{\prime} ,k}^{J}(\beta ) & = & {\sin }^{2}(\beta /2)\sqrt{\frac{(J+k)(J+k-1)}{(J-k^{\prime} )(J-k^{\prime} -1)}}{d}_{k^{\prime} +1,k-1}^{J-1}(\beta )\\  &  & +2\,\sin (\beta /2)\cos (\beta /2)\sqrt{\frac{(J+k)(J-k)}{(J-k^{\prime} )(J-k^{\prime} -1)}}{d}_{k^{\prime} +1,k}^{J-1}(\beta )\\  &  & +{\cos }^{2}(\beta /2)\sqrt{\frac{(J-k)(J-k-1)}{(J-k^{\prime} )(J-k^{\prime} -1)}}{d}_{k^{\prime} +1,k+1}^{J-1}(\beta )\end{array}$$

18 $$\begin{array}{rcl}{d}_{k^{\prime} ,k}^{J}(\beta ) & = & \sin (\beta /2)\cos (\beta /2)\sqrt{\frac{(J+k)(J+k-1)}{(J+k^{\prime} )(J-k^{\prime} )}}{d}_{k^{\prime} ,k-1}^{J-1}(\beta )\\  &  & +\,[{\cos }^{2}(\beta /2)-{\sin }^{2}(\beta /2)]\sqrt{\frac{(J+k)(J-k)}{(J+k^{\prime} )(J-k^{\prime} )}}{d}_{k^{\prime} ,k}^{J-1}(\beta )\\  &  & -\,\sin (\beta /2)\cos (\beta /2)\sqrt{\frac{(J-k)(J-k+1)}{(J-k^{\prime} )(J+k^{\prime} )}}{d}_{k^{\prime} ,k+1}^{J-1}(\beta )\end{array}$$

Despite its practicality, the method also begins to encounter some problems in precision as the quantum numbers are increased^56^.

To remedy that, Tajima^57^ has proposed a Fourier-series expansion for the matrix elements, introducing a very powerful and useful method to enhance the numerical stability and precision. Going a little beyond, Feng *et al*.^58^ presented a brilliantly simple idea to calculate the expansion coefficients by exactly diagonalizing the angular momentum operator *J*_*y*_ in the eigenbasis of *J*_*z*_. As the norm of each Fourier coefficient does not exceed unity, large-number problems in floating-point calculations are avoided, allowing us to compute the d-matrix for spins up to a few thousand with a precision of about 10^−14^ (we actually stopped at *J* = 80).

**Figure 5:**
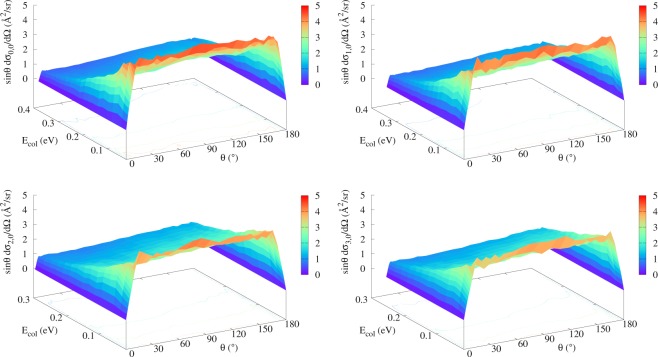
Differential cross sections (summed over all product states and multiplied by $\sin \,\theta$) as a function of the collision energy and of the scattering angle for the purely vibrational excitation of the H + Li_2_(*v*, *j* = 0) → LiH + Li reaction: (**a**) *v* = 0 (top left); (**b**) *v* = 1 (top right); (**c**) *v* = 2 (bottom left); and (**d**) *v* = 3 (bottom right). Each level curve represented differs from its predecessor by 1 Å^2^/sr.

**Figure 6:**
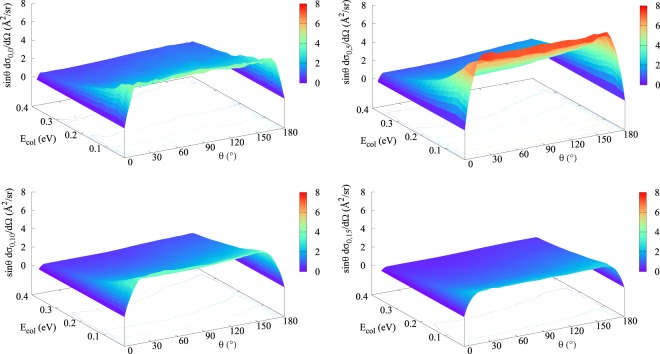
Differential cross sections (summed over all product states and multiplied by $\sin \,\theta$) as a function of the collision energy and of the scattering angle for the purely rotational excitation of the H+Li_2_(*v* = 0, *j*) → LiH+Li reaction: (**a**) *j* = 0 (top left); (**b**) *j* = 5 (top right); (**c**) *j* = 10 (bottom left); and (**d**) *j* = 15 (bottom right). Each level curve represented differs from its predecessor by 1 Å^2^/sr.

Making use of this suggestion, calculations for the differential cross sections (DCS) resulted in the graphs shown in Figs 5 and 6 (purely vibrational/rotational excitation of the reactant, respectively): same downward trend evidenced as we compare distinct panels compels us to conclude the same way as before for the integral case, with one additional analysis. Even though ref.^32^ asserts that the peaks found around *θ* = 0 and *θ* = 180° reveal that exact forward and back scatterings play a major role in the reaction dynamics, it must be underlined that in order to reach such a conclusion, the contribution of $\sin \,\theta$ ought to have been taken into consideration as well. Thus, despite having ended up with basically the same graphs of ref.^32^ at first, we multiplied the DCS by $\sin \,\theta$ and verified a nearly isotropic scenario, corresponding to an essentially flat angular distribution.

### Thermal rate coefficients

Once each execution of the ABC program yields a different (*J*, *P*, *p*)-output, containing, at the end of every single file, the *N*^*J*, *P*, *p*^(*E*) quantities that appear in Eq. (12) for the nnrg total energies starting from enrg with a dnrg step (as we set in the input parameters of Table 2), we are able to compute the cumulative reaction probabilities (CRP) by means of Eq. (11). Then, proposing a linear curve fitting for the particular points mentioned above (correlation coefficient was calculated as 0.9962319), we managed to describe *N*(*E*) at interpolation and extrapolation energies in an incredibly suitable way. As for the ro-vibrational partition function *Q*_*rovib*_ in Eq. (13), we covered all even and odd diatomic states of the Li_2_ reactant molecule obeying *E*(*v*, *j*) < 5.0 eV, thus including a total of 5885 and 5919 terms in the sum over *vj* for *p* = +1 and *p* =−1, respectively. This huge amount of states is more than enough to guarantee convergence on the denominator appearing in Eq. (14), so the upper limit of the integral plays the major role in dictating the accuracy we are dealing with.

**Figure 7:**
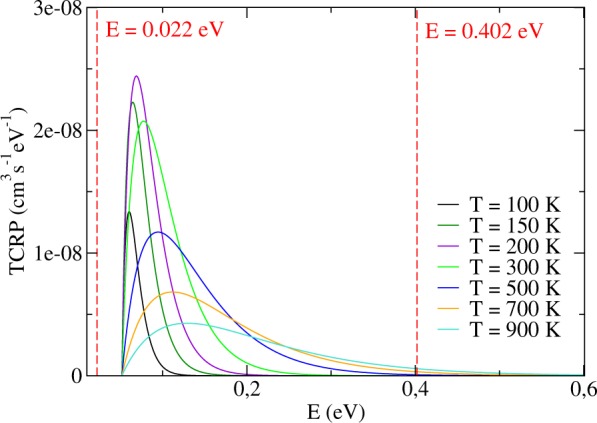
Thermal cumulative reaction probabilities as a function of the total energy.

In the case of a barrierless reaction such as the title one, where the lowest total energy at which S-matrix elements were computed is 0.022 eV, it may seem that a fictitious threshold of reactivity is being inadvertently placed at this energy, most probably impairing the correct determination of *k*(*T*), for the cross sections below that limit would implicitly be considered null. Accordingly, in order to show that no rigor was lost in the adopted procedures, thermal cumulative reaction probabilities (TCRP) were computed the way proposed in ref.^59^ and plotted in Fig. 7 for different values of temperature, what includes the minimum and maximum *T* with which we aim to work. As every contribution to the integral of the TCRP stood in the right-hand side of the red dashed line indicating *E* = 0.022 eV, the thermally averaged rate constants obtained by integration of the TCRP would have been identically those calculated following the aforementioned steps, hadn’t we decided to disregard, in the former case, the area below the curves after *E* = 0.402 eV, to better show that the CRP points before that limit sufficed to satisfactorily converge our calculations.

**Figure 8:**
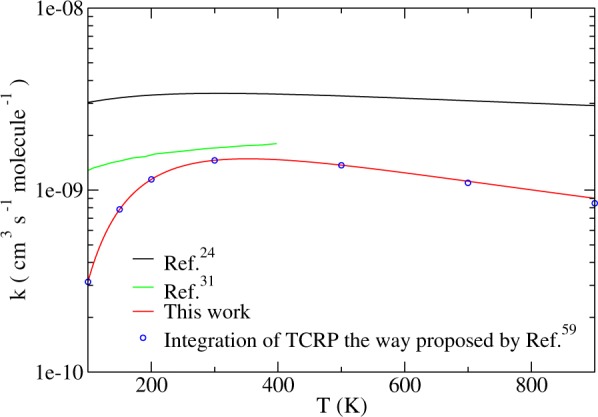
Thermally averaged rate constants for the H+Li_2_ → LiH + Li reaction revealing the discrepancy between J-shifting predictions and thorough quantum time-independent calculations.

Just to further illustrate what we mean by that, we verified that for lower temperature limits (*T* = 500 K, *T* = 700 K, and *T* = 900 K), the truncation of the TCRP’s integration at *E* = 0.402 eV was responsible for 99.7%, 98% and 94% of the final contributions to *k*(*T*), respectively. We must therefore bear in mind that the lower the temperature, the more converged are our TRC, shown in Fig. 8 together with the integration of the TCRP between *E* = 0.022 and 0.402 eV, as well as previous J-shifting^24^ and time-dependent results^31^.

From the comparison of the different curves depicted in Fig. 8, we see that, given a same PES, J-shifting approach fails here to predict the *N*^*J*,*P*,*p*^(*E*) terms based solely on the *J* = 0 behaviour the way proposed by ref.^39^ with the computation of *N*^*J*=0^ shifted from *E* by a contribution due to the geometries involved in the *J*-dependent transition states (that appear because of the addition of a centrifugal potential to the minimum energy path), *N*(*E*) had been found to be quadratic, whereas thorough quantum time-independent calculations performed in this work identified a linear response to an energy increase. For this reason, our results lay considerably lower than those derived from the application of J-shifting formalism, though once again the TRC are expected to grow up until some point in temperature (*T* ~ 350 K) and then decrease as *T* becomes higher, revealing a non-Arrhenius pattern that can also emerge from different highly exothermic reactions^60^.

## Conclusions

Thorough time-independent quantum scattering investigations were conducted in this study for the H+Li_2_ → LiH + Li reaction using the ABC program^33^ and the PES of ref.^25^, once we have developed FORTRAN codes that read the program outputs and calculate the integral and the differential cross sections, as well as the reaction TRC.

Thence, an already anticipated cross section decrease owing to an increase in energy has been spotted, a trend commonly shared by highly exothermic and barrierless reactions. Qualitative and quantitative agreement with previous theoretical works supports the good quality obtained by the application of our methodology, though experimental validation or confrontation is still pending in the literature.

Having calculated the H+Li_2_ → LiH + Li differential cross sections, we identified a nearly isotropic behaviour of the reaction, which contradicts earlier predictions from ref.^32^. Moreover, reactant purely vibrational/rotational excitation was found to hinder reactivity, a fact that is observed more intensely for the rotational case, in which we reported a significant reaction promotion that augments until *j* = 4 is reached, and then begins to retract, eventually becoming an actual inhibition for larger *j*.

Finally, our results were plotted and compared to the ones given by independent research. Even though we ended up with a similar non-Arrhenius pattern of TRC growth until ambient temperatures followed by a decrease as *T* becomes higher, the considerable discrepancy with the J-shifting approach urges us to conclude that the application of the latter most likely leads to incorrect outcome for the title reaction.

## Electronic supplementary material

Dataset 1
